# An Alternative Mechanism of Glutamate Dehydrogenase Inhibition by EGCG: Promotion of Protein Degradation

**DOI:** 10.3390/ph18060877

**Published:** 2025-06-12

**Authors:** Ziying Zeng, Chenshui Lin, Chuqiao Pan, Zhao Chen, Benfang Helen Ruan

**Affiliations:** College of Pharmaceutical Science, Collaborative Innovation Center of Yangtze River Delta Region Green Pharmaceuticals, IDD and CB, Zhejiang University of Technology, Hangzhou 310014, China; kitty-hangzhou@163.com (Z.Z.); cqpan727@163.com (C.P.); zhaochen003@outlook.com (Z.C.)

**Keywords:** EGCG, glutamate dehydrogenase, gene mutant, protein degradation, hyperinsulinism hyperammonemia syndrome

## Abstract

**Backgroud:** Glutamate dehydrogenase (GDH) is involved in the metabolism of glutamate and ammonia. It is regulated by multiple ligand variants, and hyper-active GDH mutants have been reported for hyperinsulinism hyperammonemia syndrome (HHS). **Methods:** Here, we constructed the wild-type human GDH and three human GDH454 mutants and investigated their degradation activity and performance under different GDH inhibitors. **Results:** Protein activity test and SDS-PAGE analysis of the purified proteins showed that the GDH454 mutant from HHS has weaker GDH enzymatic activity but greater resistance to trypsin hydrolysis than the wild type. Interestingly, using the biomolecular interactions technique, it showed that the GDH454 mutant has 10^9^ times weaker affinity for trypsin and 10-fold weaker for epigallocatechin gallate (EGCG) than the wild-type GDH. Subsequently, native-PAGE gel analysis demonstrated that EGCG could break down the GDH hexamer into monomers and form a complex with trypsin to enhance the degradation of both types of GDH. **Conclusions:** EGCG showed good affinity to both the wild-type and the mutant GDH proteins, promoting protein degradation; this provides a new strategy for the treatment of HHS and other hyper-active GDH-related diseases.

## 1. Introduction

Glutamate dehydrogenase (GDH), a hexameric enzyme in the mitochondrial matrix, is an important catalytic enzyme in glutamine metabolism in mammals, catalyzing the reversible hydrolysis of glutamate to produce α-ketoglutarate and ammonia, which participates in the subsequent TCA cycle. The important role of GDH in glutamine metabolism makes it a key enzyme of the carbon and nitrogen cycle in organisms; on the one hand, GDH is a key regulator of carbohydrate-induced carbon efflux; on the other hand, GDH is a key enzyme of the nitrogen cycle, mediating the deamination of glutamine into the TCA cycle [1]. In addition to an active site, human GDH has a specialized antenna structural domain that is variably regulated by a variety of small molecules, such as leucine, aspartic acid, adenine, and guanine nucleotides: GTP and ATP inhibit GDH [2], and ADP and leucine activate GDH [3].

In recent years, studies of diseases with abnormalities of GDH have demonstrated that the metabolic regulation of GDH is essential for the organism [4]. Hyperinsulinism hyperammonemia (HHS) is a rare disease caused by mutations or abnormalities in the GLUD1 gene encoding GDH, whose main clinical features are recurring episodes of symptomatic hypoglycemia and symptoms of hyperammonemia. The GLUD1 abnormality removes the inhibition of GDH by GTP either directly or indirectly, leading to an increase in the enzymatic activity of GDH. In the pancreas, dysregulated GDH increases glutamate deamination reactions, induces an overreaction of insulin to amino acid consumption, promotes ATP formation, and stimulates insulin secretion [5]; in the kidneys, hyper glutamate catabolism leads to an increase in ammonia production [6]; and in the CNS, GDH abnormalities result in a disruption of the homeostatic balance of glutamate and its derivative γ-aminobutyric acid as inter-regulatory neurotransmitters, inducing epilepsy and related conditions [7]. The 454 site of GDH is located in the antenna structural domain and belongs to the GTP-binding site, and its abnormality can impede the inhibition of GDH by GTP, thus the H454Y mutant showed hyper-GDH-activity and could induce typical HHS damage [8].

**Figure 1:**
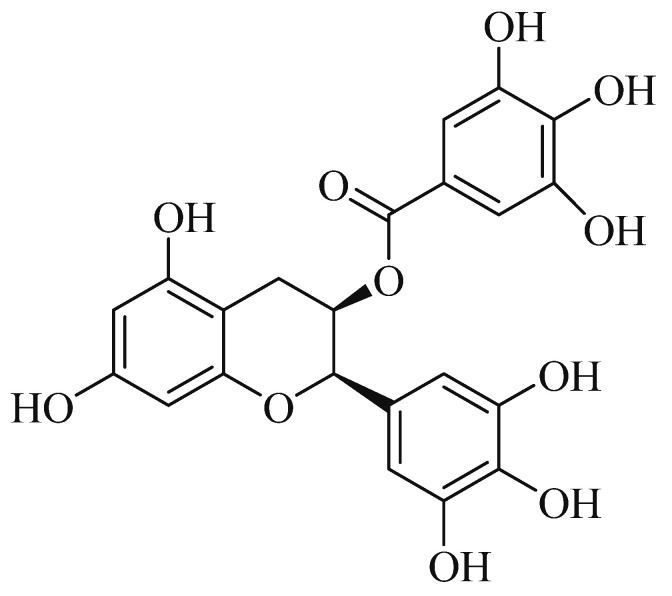
Chemical structural diagram of EGCG.

The main GDH inhibitors reported so far are epigallocatechin, chlorsulfuron-containing biothiols with GW5074, selegiline, etc. Other types of GDH inhibitors bind to GDH at different binding sites. EGCG, a phenol derivative, can inhibit the mitochondrial enzyme glutamate dehydrogenase in the micromolar range [9]. EGCG, a naturally occurring active substance extracted from green tea taxol [10], has been shown to inhibit the mitochondrial enzyme glutamate dehydrogenase in tea polyphenols. EGCG is a naturally active substance extracted from green tea polyphenols. Among tea polyphenols, catechins have the highest content, including (-)-epigallocatechin (EC), (-)-epigallocatechin (EGC), (-)-epigallocatechin-3-gallate (CG), and (-)-epigallocatechin-3-gallate (EGCG) [11], with the highest concentration of EGCG (accounting for 50% to 70% of the proportion of tea polyphenols), and the most potent biological activity [12]. A large number of studies have shown that EGCG has beneficial biological activities in antioxidant [13], antibacterial [14], anti-obesity, anti-inflammatory, anti-tumor, cardiovascular protection, neuroprotective activities [15], and other aspects of health, and EGCG compared with other polyphenolic compounds have stronger biological activity, which is closely related to its molecular structure [16]. Epigallocatechin gallate (EGCG, chemical name (−)-cis-2-(3,4,5-trihydroxyphenyl)-3,4-dihydro-1(2H)-benzopyran-3,5,7-triol 3-gallate) (Figure 1) is a polyphenolic derivative belonging to the catechin family, consisting of a flavan-3-ol portion linked to gallic acid to form an ester group. The large number of hydroxyl groups on the aromatic ring and their unique distribution make EGCG have superior biological activities.

The 8 phenolic hydroxyl structure of EGCG makes it extremely hydrophilic, which makes its interaction with GDH mainly polar and binds to GDH at the ADP binding site [17]. Unlike other inhibitors, EGCG can bind to GDH in the closed conformation, and even at high concentrations of Glu and NADPH, EGCG still seems to push the GDH structure into the open conformation [18]. Unlike ADP, which inhibits the reaction under conditions where product release is not a rate-limiting step (at low substrate or cofactor concentrations), EGCG inhibits GDH under all conditions [19]. There are two possible explanations for this inhibition. The first explanation is that EGCG affects the overall Gibbs free energy of the protein. The ED50 of EGCG with GDH is lower than that of other inhibitors, suggesting that it reduces the Gibbs free energy of the protein, making conformational changes of the GDH protein more difficult. In this case, the GDH open conformation is the conformational state with the lower required capacity. This suggests that the inhibition of GDH by EGCG may not be achieved through dependence on specific interactions but rather is related to an increase in affinity for the protein and a decrease in the protein energy state. The second explanation is that EGCG alters the overall energy state of the enzyme. It was shown that the binding of EGCG to GDH did not increase the agonistic effect on GDH even when the binding affinity of the ADP analog to GDH was increased. This suggests that it is more likely that EGCG inhibits GDH primarily by altering the overall energy state of the enzyme rather than by specific interactions [9].

To further understand the role of GDH mutant in HHS and seek new treatment methods, we explored the mechanism of action using the wild-type GDH, the cloned and expressed hGDH_H454Y_ protein, and active site binder EGCG. Previous studies [8] have shown that the GDH_H454Y_ mutant has a significantly lower binding affinity for GTP than wild-type GDH, resulting in a weakened ability for GTP to inhibit GDH mutant in HHS patients. Our study showed that the hGDH_H454Y_ mutant exhibits significantly reduced binding capacity to trypsin compared to wild-type hGDH, thereby interfering with GDH protein degradation in HHS patients. This finding provides another explanation for the persistent overexpression of GDH in HHS patients. Additionally, our study demonstrated that the GDH inhibitor EGCG not only directly inhibits GDH activity but also disrupts the hexameric structure of GDH proteins, promoting their hydrolysis, which provides new insights for targeting GDH mutants in HHS disease.

**Figure 2:**
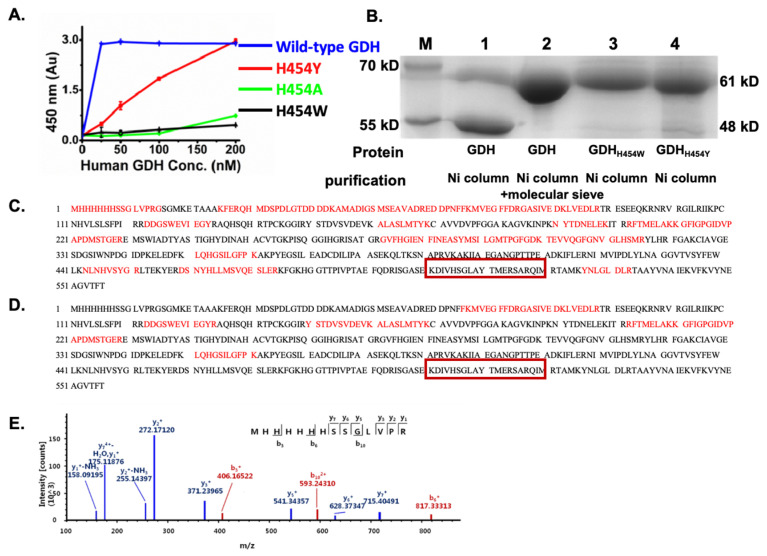
The wild-type hGDH and hGDH_H454Y_ cloned and expressed in *E. coli*. (**A**) Dose–response of the purified wild-type and mutant hGDH_H454Y_ proteins shows the enzyme activity. (**B**) SDS gel analysis of the wild type and the mutant hGDH_H454Y_ proteins expressed in *E. coli* and purified by nickel affinity chromatography. (1) Human wild-type GDH; (2) Human wild-type GDH further purified by molecular sieve; (3) Human GDH_H454W_; (4) Human GDH_H454Y_. (**C**) The sequences marked in red are the fragments found in the MS analysis of the 61 kDa protein, corresponding to the full-length wild-type hGDH; the boxed sequence is the hGDH_H454_ mutation site; (**D**) The sequences marked in red are the fragments found in the MS analysis of the 48 kDa protein, corresponding to the C-terminal truncated hGDH; (**E**) MS of the N-terminal His-tagged fragment found in all the tested 61 kDa proteins (the wild-type or mutant GDH proteins).

**Figure 3:**
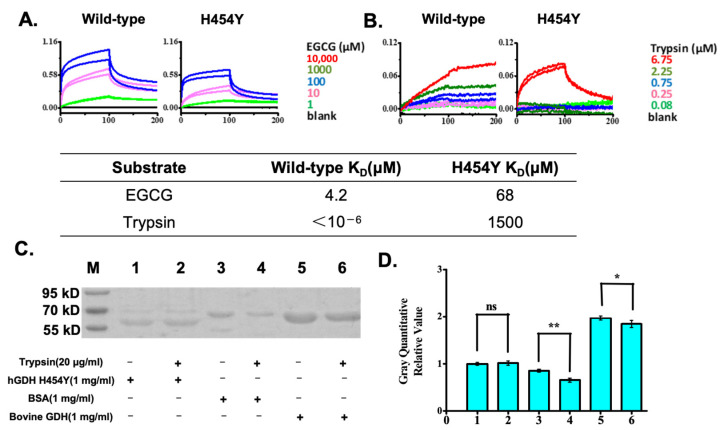
Biolayer interference-based substrates binding to hGDH assay. (**A**) EGCG binds well to both the wild-type hGDH and hGDH_H454Y_ with an estimated K_D_ 4.2 μM and K_D_ 68 μM, respectively; (**B**) Trypsin binding to the wild-type hGDH tightly with very small off-rate and with an estimated K_D_ < 10^−6^ μM, whereas hGDH_H454Y_ showed a much weaker binding with an estimated K_D_ 1500 μM.; (**C**) Gel analysis of the 1 mg/mL hGDH_H454Y_ (band 1–2), BSA (band 3–4) and bovine GDH (band 5–6) treated with 20 μg/mL trypsin (band 2, 4, 6) or without trypsin (band 1, 3, 5). (**D**) Normalized hGDH_H454_, BSA, and bovine GDH protein concentration in the presence or absence of trypsin degradation. The experiments were repeated three times. Statistical analysis was performed via a *t*-test: * *p* < 0.05, ** *p* < 0.01. n. s., no significant.

**Figure 4:**
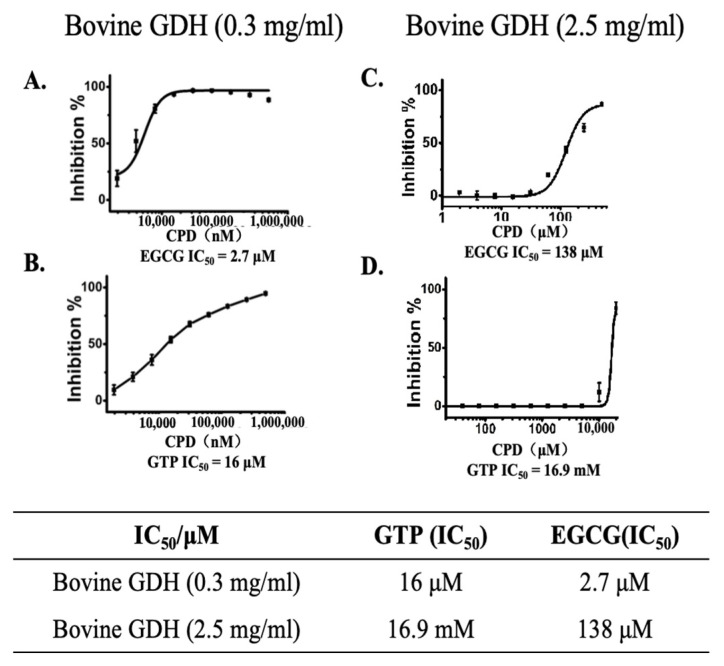
Inhibitory effect of EGCG is less affected by the GDH enzyme concentration than that of GTP. (**A**,**B**) Inhibition of low-concentration bovine GDH (0.3 mg/mL) by EGCG and GTP; (**C**,**D**) Inhibition of high-concentration bovine GDH (2.5 mg/mL) by EGCG and GTP.

**Figure 5:**
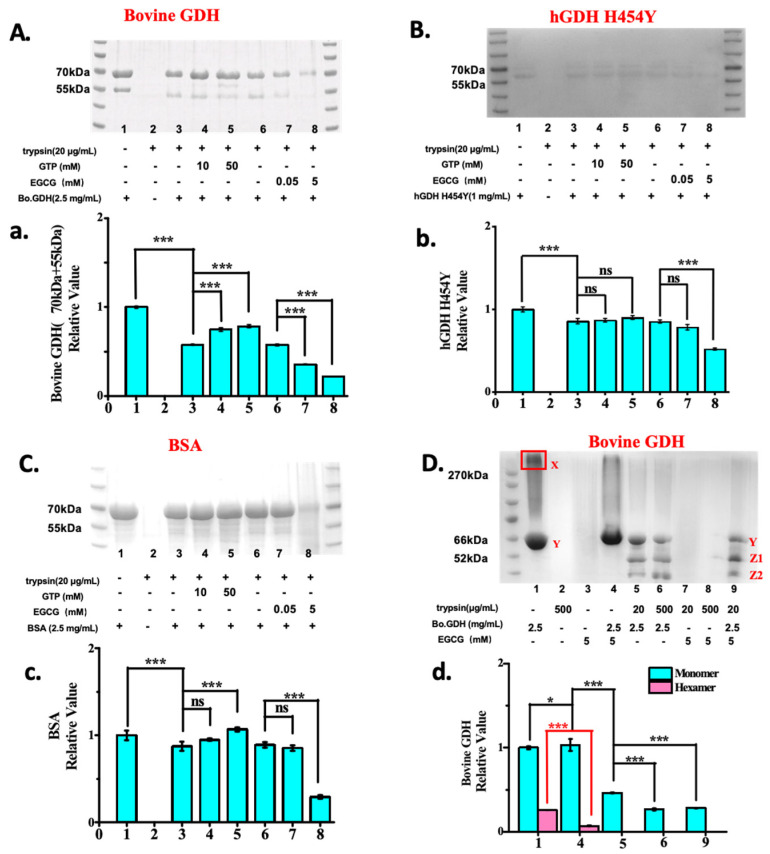
Trypsin hydrolysis of GDH, hGDH_H454Y_ and mechanistic studies. (**A**,**a**) Effect of EGCG on trypsin digestion of bovine GDH. (**B**,**b**) Effect of EGCG on trypsin digestion of hGDH_H454Y_. (**C**,**c**) Effect of EGCG-trypsin complex on trypsin digestion of BSA. (**D**,**d**) Interaction of EGCG, bovine GDH and trypsin analyzed by native-PAGE gels. X: The hexamers amount of bovine GDH; Y: The monomers’ amount of bovine GDH in the presence of EGCG or not. Z1, Z2: The hydrolyzed products of bovine GDH by trypsin in the presence of EGCG or not. Statistical analysis was performed via a *t*-test: * *p* < 0.05, *** *p* < 0.001. n. s., no significance.

It is worth stating that our comparative analysis based on the NCBI database revealed that the amino acid sequence similarity between bovine glutamate dehydrogenase (Bovine GDH) and human GDH (hGDH) was as high as 98%, with almost identical substrate sites and regulatory sites. Therefore, Bovine GDH is an excellent substitute for human GDH. In addition, in the process of protein purification, it is often affected by some interfering factors (e.g., temperature, pH, reagents, etc.).,So we used hGDH as the research object in the cloning and protein purification, detection of the enzymatic activity of the purified protein, and molecular interaction experiments, the results of which are displayed in Figure 2 and Figure 3A,B. We chose commercially available Bovine GDH as the experimental subject when we investigated the digestion of different proteins by trypsin, as well as the effects of the inhibitors EGCG and GTP on the activity of proteins, and the diagrams involved are Figure 3C,D, Figure 4 and Figure 5.

## 2. Results

### 2.1. The GDH H454 Mutants Expressed in E. coli Showed Weaker Enzyme Activity and Greater Resistance to Proteolysis Compared to the Wild-Type

Firstly, we tried to construct different types of GDH H454 mutants and compared their activities. The N-terminal His-tagged hGDH mutants (hGDH_H454Y_, hGDH_H454A_, and hGDH_H454W_) were successfully cloned and expressed in *E. coli.* The proteins were purified by nickel affinity chromatography, and the enzymatic activity was determined by the GDH activity assay based on the EZMTT (2-(3-(2-methoxy-4-nitrophenyl)-2-(4-nitrophenyl)-2H-tetrazol-3-ium-5-yl) benzenesulfonate sodium salt method [20]; the principle is that GDH can catalyze the conversion of L-glutamic acid (Glu) to α-ketoglutaric acid (α-KG) and NH_3_ in the presence of either nicotinamide adenine dinucleotide (NAD^+^) or nicotinamide adenine dinucleotide phosphate (NADP^+^). As shown in Figure 2A, in comparison to the wild-type hGDH, the enzyme activity of the mutants (hGDH_H454Y_, hGDH_H454A_, and hGDH_H454W_) was significantly decreased; after 4 h incubation, the mutant hGDH_H454Y_ showed moderate catalytic activity, which was about 8-times lower than that of the wild-type, whereas the mutant hGDH_H454A_ and hGDH_H454W_ essentially showed no activity. Since only the hGDH_H454Y_ retained its activity, we used the mutant hGDH_H454Y_ as the model protein for the rest of the research.

Interestingly, the homozygous hGDH_H454Y_ showed lower enzymatic activity, but at the protein level, the SDS gel experiments demonstrated that hGDH_H454Y_ appeared to be more resistant to protease degradation than the wild-type GDH proteins. As shown in Figure 2B, after Ni column purification, the wild-type hGDH showed two bands with molecular weights of 61 kDa and 48 kDa, whereas hGDH_H454Y_ showed only one band with molecular weight of around 60 kDa. Therefore, the apparent enhanced cellular GDH activity might have resulted from the prolonged existence of hGDH_H454Y_.

Further proteomic analysis showed that the 60–61 kDa bands contained the N-terminal His-tagged fragment (Figure 2C,E), confirming that these are the full-length wild-type hGDH (Figure 2B, No. 2 band), hGDH_H454W_ (Figure 2B, No. 3 band), or hGDH_H454Y_ proteins (Figure 2B, No. 4 band), respectively. On the other hand, the 48 kDa proteins were determined as the C-terminal domain cleaved wild-type hGDH (Figure 2B, No. 1 lower band) because the MS fragments (in red) beyond the H454 residue could not be found in the MS analysis as shown in Figure 2D. Since none of the hGDH_H454_ mutants showed protein degradation, we hypothesized that hGDH_H454_ mutation might prevent protease docking at the C-terminal, which is responsible for inhibiting proteolysis, although the mechanism has not been reported.

### 2.2. Biomolecular Interaction Analysis Demonstrated That the hGDH_H454Y_ Mutant Greatly Reduced Its Affinity to a Protease, While EGCG Bound Well to Both GDHs

The wild-type GDH and the hGDH_H454Y_ mutant possess different enzymatic activities and protease stability. The proteomics results in Figure 2 suggested that the H454 mutation may be a key factor affecting protease binding to GDH, and the hGDH_H454_ mutation may prevent the protease docking at the C-terminus. We further tested EGCG binding to both the wild-type and mutant GDH by biomolecular interaction assays.

After immobilization of His-tagged wild-type GDH or hGDH_H454Y_ (20 μg/mL) on a Ni-NTA biosensor, the probe was immersed in a series of dilutions of EGCG to measure binding. As shown in Figure 3A, the inhibitor EGCG could bind to both wild-type and hGDH_H454Y_, although EGCG showed a 17-fold stronger affinity (K_D_) for the wild-type GDH than for the H454Y mutant.

When trypsin, an enzyme with a wide range of hydrolytic capabilities, was tested, we observed that, as shown in Figure 3B, the affinity (K_D_) of trypsin for the hGDH_H454Y_ decreased by 9 orders of magnitude (10^9^ folds) in comparison to that of the wild-type GDH, indicating that wild-type GDH could be readily recycled and regulated by protein degradation, whereas the hGDH_H454Y_ may not be readily hydrolyzed in cells. This might explain why the hGDH_H454Y_ protein showed enzymatic activity but was reported to have hyper-GDH activity; the accumulation of low-functioning mutants may account for the increase in overall GDH activity, but the abnormal accumulation might further interfere with various biological activities.

To confirm the variability and specificity of trypsin digestion, hGDH_H454Y_, BSA, and bovine GDH (wild-type GDH) were selected for a brief 1 h trypsin digestion, as shown in Figure 3C,D. Interestingly, 20 μg/mL trypsin did not show observable degradation of the hGDH_H454Y_ (bands No. 1 and 2) within 1 h, whereas both BSA and bovine GDH showed significant hydrolysis. This is consistent with the protein degradation results in Figure 2 and further indicates that the observed high cellular GDH activity of the hGDH_H454Y_ might have resulted from a decrease in the affinity of intracellular proteases for the H454Y mutation, preventing its hydrolysis, and resulting in sustained overexpression. If the hypothesis is true, we might be able to provide a new therapeutic approach for HHS by inducing protein degradation for GDH mutants.

### 2.3. EGCG Is a Better Allosteric GDH Inhibitors than GTP and Promotes Protease Degradation of Both Wild-Type and hGDH_H454Y_ Proteins

Even though the wild-type and hGDH_H454Y_ proteins differ significantly in terms of trypsin hydrolysis, EGCG showed a relatively good binding affinity for both proteins (Figure 3A,B). Since both GTP and EGCG are known allosteric GDH inhibitors, we tested their dose-dependent inhibition of different levels of GDH protein. As shown in Figure 4, under the optimized assay conditions, at 0.3 mg/mL GDH concentration, the IC_50_ values of EGCG and GTP were 2.7 μM and 16 μM, respectively, whereas at 2.5 mg/mL GDH, the values increased to 138 μM and 16.9 mM, respectively. Interestingly, increasing GDH concentration by 8 times could result in an increase in the IC_50_ values of EGCG and GTP by 50-fold and 1000-fold, respectively. EGCG inhibition of GDH is less affected by the GDH enzyme concentration.

Further, to test the specificity and mechanism of EGCG-mediated GDH degradation, we used gel analysis to explore the influence of the presence or absence of high or low levels of EGCG or GTP on trypsin digestion of Bo.GDH, hGDH_H454Y,_ and BSA proteins. As shown in Figure 5A,a, both low and high concentrations of GTP prevented the trypsin digestion of Bo.GDH, whereas EGCG greatly increased trypsin digestion in a dose-dependent manner with a statistically significant *p*-value (*p* < 0.001). Interestingly, as shown in Figure 5B,b, when investigating the effect of GTP and EGCG on trypsin digestion to hGDH_H454Y_ protein, GTP showed no effect on hGDH_H454Y_ hydrolysis by trypsin, even at a high concentration (50 mM). However, 5 mM EGCG was able to promote trypsin’s hGDH_H454Y_ hydrolysis (lane 8 in Figure 5B), which is similar to the wild-type results.

The trypsin–EGCG complex has been reported [21,22], but the function of this complex has not been elucidated. Based on our results, we speculated that the mechanism of action of the trypsin–EGCG complex is due to its formation of a trypsin–EGCG complex with various target proteins. When BSA was tested (Figure 5C,c), at lower concentrations of GTP (10 mM) and EGCG (0.05 mM), both compounds had essentially no significant effect on trypsin digestion. However, 50 mM GTP prevented BSA digestion, which was statistically significant, whereas 5 mM EGCG promoted BSA degradation, although protein precipitation was observed in the 5 mM EGCG-BSA digestion assay. In particular, the finding that EGCG promotes the degradation of the hGDH_H454Y_ mutant suggests that EGCG-induced degradation of hGDH mutants for the treatment of HHS disease may be able to be a new therapeutic strategy.

To further understand the mechanism of EGCG promoting GDH protein degradation, we investigated the interactions among EGCG, GDH, and trypsin by native gel analysis. Because the bovine GDH protein sequence shares 98% homology with the human GDH sequence and 100% homology similarity in the substrate and regulatory binding sites, we used bovine GDH as a substitute for wild-type human GDH in our studies. Figure 5D,d shows that GDH exists as a hexamer in the absence of EGCG or trypsin (boxed band in Lane 1). However, in the presence of 5 mM EGCG or trypsin (20–500 μg/mL), the hexamers of bovine GDH were converted to monomers (Lane 4, 5, 6), and in addition, monomeric GDH was degraded by trypsin in a dose-dependent manner (Lane 5 and 6). If 5 mM EGCG was added to the mixture of GDH and 20 μg/mL trypsin (Lane 9), more monomeric GDH degradation was observed (Lanes 5, 9). Taken together, the mechanism might be the following: binding of EGCG to hexametric GDH breaks down the hexamer into monomers, and further EGCG–trypsin complex facilitates the GDH degradation.

## 3. Discussion

GDH is a mitochondrial enzyme in mammalian cells that catalyzes the reversible reaction of glutamate deamination to produce alpha-ketoglutarate, which has a crucial role in maintaining glutamate levels and ammonia metabolism. Most cases of HHS are due to heterozygous missense mutations in the GLUD1 gene, which encodes GDH, resulting in hypoglycemia and hyperammonemia, usually with onset in infancy and childhood [23,24]. Currently, only symptomatic treatment is available in clinics [25]. Diazoxide can significantly improve the hypoglycemic symptoms of HHS patients [26], but it cannot effectively control hyperammonemia and some neurological damage in the patients because its target of action is the K_ATP_ channel and not the GDH or glutamate metabolic pathway. Therefore, there is an urgent need to develop a drug that can improve the overexpression of GDH and treat HHS from the perspective of addressing the pathogenesis. EGCG is a catechin derived from green tea polyphenols, is an allo-inhibitor of GDH, and has been demonstrated to have multiple biological activities such as antioxidants, antimicrobial, and antitumor. H. Smith et al. [27] showed that EGCG could effectively inhibit not only wild-type GDH but also GDH mutants in HHS, suggesting that EGCG has the potential to treat HHS.

Previous studies have suggested that GDH in patients with HHS has a gain of function mutation by weakening the inhibitory activity of GTP or interfering with the GTP/ADP regulation process [28]. However, the homogenous hGDH_H454Y_ protein we purified showed decreased enzyme activity. GDH proteins in HHS patients exist as heterohexamers, but it is unlikely that a mixture of wild-type and hGDH_H454Y_ mutant proteins would be regulated by ATP/ADP to acquire GDH function. SDS-PAGE gel analysis during protein purification showed two bands at 61 kDa and 48 kDa for wild-type hGDH (Figure 2B, lane 1), while the GDH_H454_ mutant showed only a single intact band at 61 kDa (Figure 2B, lane 2–4). Protein mass spectrometry identification confirmed that the 48 kDa fragment corresponds to the C-terminal residues H454-L505 of GDH, which contains trypsin cleavage sites (R457 and R483). This revealed that the missing 15 KDa peptide was located at the C-terminus of the wild-type GDH protein, including the H454 site. This suggests that the degradation of wild-type GDH may have happened during purification and that the mutation at position 454 prevented this degradation. Subsequently, we discovered 10^9^ orders of magnitude differences in trypsin binding between the wild-type and mutant GDH_H454_ protein, and wild-type hGDH could be degraded by trypsin while hGDH_H454Y_ could not be degraded. This suggests that wild-type GDH can be regulated by protein degradation, whereas mutant GDH_H454Y_ may remain in the biological system for a longer period due to the weakened ability of degradation. Although functional activation due to mutations in GDH has often been implicated as a pathogenic mechanism for HHS, our study demonstrates that GDH 454 mutations make degradation by proteolytic enzymes difficult; thus, continuation of the performance of its biological function in the patient is also difficult, representing an additional pathogenic cause of HHS.

The problem in HHS patients is not only the hyperfunction of GDH; the strong resistance to protease degradation exhibited by the H454Y mutant makes their removal difficult. Intracellular protein degradation is a major metabolic activity with a protein quality control function and an important regulatory role. In vivo, GDH can become protease-sensitive due to the loss of stabilizing ligands or interaction with unstable metabolites accumulated in starved cells [29]. Furthermore, any of several adenosine triphosphate (ATP)-dependent proteases could degrade sensitive proteins [30]. Therefore, since thermal and protein hydrolytic stability are important for GDH activity [30], the presence of protein degradation resistance would significantly affect GDH function. Fortunately, our research shows that EGCG, as an allosteric inhibitor of GDH, has a more potent direct inhibitory effect on GDH than GTP, and enhances the degradation of GDH by trypsin, which is not possible with other GDH inhibitors such as GTP. EGCG promotes the hydrolysis ability of trypsin to GDH protein, whereas GTP inhibits the hydrolysis of GDH. After binding with trypsin, EGCG disrupts the hexameric structure of GDH, converting it into monomers, and significantly promotes the degradation of GDH by trypsin. (*** *p* < 0.001). Our results suggest that inducing degradation of hGDH mutants by EGCG may be a potential approach for the treatment of HHS patients.

The study established the reliability of the conclusions through multi-method cross-validation, rigorous statistical design, and independent replicate experiments. All experiments were validated through repeatability testing, with all gel experiments repeated three times using independently prepared samples. All key conclusions were derived through rigorous statistical analysis, and all statistically significant differences were confirmed via *t*-tests (*p* * < 0.05).

Our studies have demonstrated that EGCG enhances the ability of trypsin to hydrolyze both wild-type and GDH_H454Y_ mutants, but further studies are needed to fully understand the details of the structural changes in the GDH hexamer upon binding of the EGCG–trypsin complex to the GDH protein. Although our research has demonstrated that EGCG can break down GDH hexamers into monomers, further verification is needed to confirm whether this effect is unique to EGCG.

## 4. Materials and Methods

### 4.1. Materials

*E. coli* BL21 (DE3) and DH 5α strain were purchased from Vazyme (Nanjing, China). Mut Express Ⅱ Fast Mutagenesis Kit V2 (C214-01/02) was purchased from Vazyme and pG-KJE8 vector was purchased from YouBio (Changsha, China). The protein concentration was measured using a spectrophotometer (BioDrop). Ni-NTA agarose was purchased from Qiagen (Venlo, The Netherlands). Glutamate was purchased from Biosharp, Inc. (Hefei, China). EGCG, glycine, skim milk, α-KG, NAD, and β-NADP were purchased from Shanghai Yuanye Bio-Technology Co., Ltd. (Shanghai, China). Chloramphenicol and kanamycin were purchased from Shanghai Jaming Biotechnology Co., Ltd. (Shanghai, China). The protease and phosphatase inhibitor mini-tablets were purchased from Sigma(Darmstadt, Germany). NP40 lysis buffer was purchased from GenStar (Beijing, China). SDS was purchased from Xilong Chemical Co., Ltd. (Shantou, China). 40% (*v*/*v*) acrylamide/Bis solution was purchased from Beijing Solarbio Science & Technology Co., Ltd. (Beijing, China). The 5×SDS sample buffer was purchased from KingsRui Biotechnology Co., Ltd. (Nanjing, China). The EZMTT detection reagents were obtained from JNF Bioscience LLC. (Hangzhou, China). Penicillin-streptomycin was purchased from Beijing Solarbio Science & Technology Co., Ltd. (Beijing, China). Diazoxide was purchased from MCE (Shanghai, China). Bovine serum albumin (BSA) was purchased from Biosharp. The plasmid miniprep kit was purchased from Axygen (Silicon Valley, CA, USA). The Pall Fortebio Octet^®^ K2 was purchased from ForteBio, Inc. (Silicon Valley, CA, USA), and the biosensors were purchased from Pall Fortebio, Inc. The microplates were supplied by Greiner Bio-One, Inc. (Frickenhausen, Germany). Flexstation 3 was purchased from Molecular Device (Silicon Valley, CA, USA).

### 4.2. Cloning and Purification of the Human GDH Mutants

Human GDH (GLUD1: NM_005271.4)/pET-30a recombinant plasmid was constructed, as described previously [31]. We created three mutations, H454Y (cDNA substitution: C1359→T), H454W (CAC1359-1362→TGG), and H454A (CAC1359-1362→GCA) using Mut Express Ⅱ Fast Mutagenesis Kit V2. All the constructions were confirmed by DNA sequencing. The plasmid was transformed into an *E. coli* BL21 codon plus strain for protein expression. The recombinant mutant hGDH proteins were expressed after 0.5 mM isopropyl beta-D-thiogalactopyranoside (IPTG) induction for 3 h and purified using nickel affinity chromatography. The purified protein was analyzed by SDS-PAGE, which showed a single band with a molecular weight ranging between 45 kDa and 65 kDa; it was then stored in buffer (20 mM Hepes, 200 mM NaCl).

### 4.3. Activity Assay of the Human GDH Mutants

Two-fold serial dilutions of the purified wild-type and mutant human GDH (100, 50, 25, 12.5, 6.2, 3.1, 1.6 and 0 nM) were made in a 96-well plate in reaction buffer (50 mM Tris-HCl, 0.01% (*v*/*v*) BSA, 0.003% (*v*/*v*) Brij-35, 0.001% Tween 20, pH 8.0), followed by addition of a mixture of Glu (10 mM final), NADP+ (200 μM final), and the EZMTT detection reagent to each well. The reaction was carried out at room temperature before the absorbance measurement at 450 nm.

### 4.4. Biomolecular Interaction Assay

The inhibitor/substrate binding assays were performed in 3 steps: step one is coating the Ni-NTA biosensor with the human wild type and the mutant GDH proteins (20 μg/mL) for 30 min; step two is dipping the coated individual sensor into a series dilution of substrates or inhibitors, such as EGCG (0, 1, 10, 100, 1000, 10000 μM; 10-fold dilution) or trypsin (0, 0.08, 0.25, 0.75, 2.25, 6.75 μM; 3-fold dilution) for 100 s, to obtain the on-rate (kon); step three is dipping to wash with phosphate-buffered saline (PBS) for 100 s. to measure the off-rate (koff). The KD was calculated by dividing the kon with Koff.

### 4.5. GDH Inhibition Assay

Dilutions of inhibitors (0–13 μM; 3-fold dilutions) were spotted in 96-well plates and mixed with wide-type human GDH or mutation human GDH in buffer (50 mM Tris-Cl, 0.01% (*v*/*v*) BSA, 0.001% (*v*/*v*) Tween 20, pH 8.0). After 0.5 h pre-incubation, a mixture of Glu (10 mM final), NADP^+^ (200 mM final), and the EZMTT detection reagent were added to each well, and the reaction was carried out at room temperature for 1 h before the absorbance measurement at 450 nm. To exclude assay artifacts, controls with no GDH were also used, and all experiments were carried out in triplicate and repeated 3 times.

### 4.6. Trypsin Degradation Assay of Bovine GDH, hGDHH454Y and BSA

Trypsin (final concentration 20 μg/mL) were mixed with 1 mg/mL (final concentration) hGDH-H454Y or bovine-GDH or BSA, respectively, in Eppendorf (EP) tubes in a phosphate buffer (pH8; 10 mM Na_3_PO_4_, 50 mM K_3_PO_4_, 100 mM NH_4_HCO_3_, 0.1% Tween80 and 0.1% Tween). Then proteins were subjected to digestion in a wet chamber at 37 °C for 1 h. Each sample was analyzed by denaturing SDS-PAGE gel. All experiments were repeated 3 times.

### 4.7. Trypsin Degradation Assay of Bovine GDH and BSA in the Presence of EGCG and GTP

2.5 mg/mL bovine GDH (final concentration) or 2.5 mg/mL BSA proteins were mixed with various compounds (10 mM or 50 mM GTP; 0.05 mM or 5 mM EGCG) in EP tubes at room temperature for 1 h. To each tube, 20 μg/mL trypsin was added, and the reaction was carried out in a wet chamber at 37 °C for 1 h. Samples were analyzed by both denaturing SDS-PAGE gel and native-PAGE gel. All experiments were repeated 3 times.

### 4.8. Gel Analysis and Proteomic Analysis

The human wild type and the mutant GDH proteins were purified by nickel affinity chromatography. The proteins were separated by SDS-PAGE, Coomassie-stained, and destained with a solution containing 25 mM NH_4_HCO_3_ and 50% (*v*/*v*) acetonitrile (ACN).

For the protein of interest, the protein band was cut from SDS-PAGE gel, chopped into small pieces, and subjected to trypsin digestion in a wet chamber at 37 °C overnight. After digestion, the solution was acidified with 1% (*v*/*v*) trifluoroacetic acid (TFA) for desalting. Desalting was carried out using C18 columns. The C18 columns were activated by two rinses with 50% (*v*/*v*) ACN and then equilibrated by two rinses with 0.1% (*v*/*v*) TFA solution. Subsequently, the acidified peptide solution was slowly aspirated and dispensed by the activated C18 tip for 10 cycles. Desalting was conducted through two washes with 0.1% (*v*/*v*) TFA solution. The desalted peptides were eluted using 50% (*v*/*v*) ACN (50 μL), and the elution product was dried under vacuum and resuspended in 0.1% (*v*/*v*) TFA (10 μL).

### 4.9. Gel Strip Analysis Assay

For all Native-PAGE and SDS-PAGE covered in the text, the following analysis assay was used:

The dried gel was placed in the imager and photographed after the bands were clear. The gray value of the bands was calculated on a uniform background using Image-J software (1.53a). The ratio of the gray value of the strips under each experimental condition to the gray value of the control strips was calculated, as it is the relative gray value of the strips under that experimental condition.

The results of the analysis for the gel bands in this study are all presented in the form of relative gray values.

### 4.10. Statistical Analysis Assay

The experiments set up in this study were repeated three times, the mean and standard deviation were calculated, and the *p*-value between the two groups of data was calculated by GraphPad Prism 8 to compare whether there was a significant difference between the groups, i.e., *: *p* < 0.05, **: *p* < 0.01, ***: *p* < 0.001.

## Data Availability

The original contributions presented in this study are included in the article. Further inquiries can be directed to the corresponding author(s).

## References

[1] Pournourmohammadi S., Grimaldi M., Stridh M.H., Lavallard V., Waagepetersen H.S., Wollheim C.B., Maechler P. (2017). Epigallocatechin-3-gallate (EGCG) activates AMPK through the inhibition of glutamate dehydrogenase in muscle and pancreatic ß-cells: A potential beneficial effect in the pre-diabetic state?. Int. J. Biochem. Cell Biol..

[2] Jin Y., Li D., Lu S., Zhao H., Chen Z., Hou W., Ruan B.H. (2018). Ebselen Reversibly Inhibits Human Glutamate Dehydrogenase at the Catalytic Site. Assay Drug Dev. Technol..

[3] Zhao Y., Gao J., Su S., Shan X., Li S., Liu H., Yuan Y., Li H. (2021). Regulation of the activity of maize glutamate dehydrogenase by ammonium and potassium. Biosci. Biotechnol. Biochem..

[4] Pan C., Mao S., Xiong Z., Chen Z., Xu N. (2023). Glutamate dehydrogenase: Potential therapeutic targets for neurodegenerative disease. Eur. J. Pharmacol..

[5] Zeng Q., Sang Y. (2023). Glutamate dehydrogenase hyperinsulinism: Mechanisms, diagnosis, and treatment. Orphanet. J. Rare Dis..

[6] Valdivielso J., Eritja A., Caus M., Bozic M. (2020). Glutamate-Gated NMDA Receptors: Insights into the Function and Signaling in the Kidney. Biomolecules.

[7] Rosenfeld E., Nanga R., Lucas A., Revell A.Y., Thomas A., Thomas N.H., Roalf D.R., Shinohara R.T., Reddy R., Davis K.A. (2022). Characterizing the neurological phenotype of the hyperinsulinism hyperammonemia syndrome. Orphanet. J. Rare Dis..

[8] Nassar O., Li C., Stanley C., Pettitt B.M., Smith T.J. (2019). Glutamate dehydrogenase: Structure of a hyperinsulinism mutant, corrections to the atomic model, and insights into a regulatory site. Proteins.

[9] Li M., Li C., Allen A., Stanley C.A., Smith T.J. (2012). Glutamate Dehydrogenase: Structure, Allosteric Regulation, and Role in Insulin Homeostasis. Neurochem. Res..

[10] Xing L., Zhang H., Qi R., Tsao R., Mine Y. (2019). Recent Advances in the Understanding of the Health Benefits and Molecular Mechanisms Associated with Green Tea Polyphenols. J. Agric. Food Chem..

[11] Peng X., McClements D., Liu X., Liu F. (2024). EGCG-based nanoparticles: Synthesis, properties, and applications. Crit. Rev. Food Sci. Nutr..

[12] Khan N., Mukhtar H. (2018). Tea Polyphenols in Promotion of Human Health. Nutrients.

[13] Yokotani K., Umegaki K. (2017). Evaluation of plasma antioxidant activity in rats given excess EGCg with reference to endogenous antioxidants concentrations and assay methods. Free Radic. Res..

[14] Lee S., Al G., Kwon D. (2017). Antibacterial activity of epigallocatechin-3-gallate (EGCG) and its synergism with β-lactam antibiotics sensitizing carbapenem-associated multidrug resistant clinical isolates of Acinetobacter baumannii. Phytomed. Int. J. Phytother. Phytopharm..

[15] Eng Q., Thanikachalam P., Ramamurthy S. (2018). Molecular understanding of Epigallocatechin gallate (EGCG) in cardiovascular and metabolic diseases. J. Ethnopharmacol..

[16] Nikoo M., Regenstein J., Gavlighi H. (2018). Antioxidant and Antimicrobial Activities of (-)-Epigallocatechin-3-gallate (EGCG) and its Potential to Preserve the Quality and Safety of Foods. Compr. Rev. Food Sci. Food Saf..

[17] Chang S., Keretsu S., Kang S. (2022). Evaluation of decursin and its isomer decursinol angelate as potential inhibitors of human glutamate dehydrogenase activity through in silico and enzymatic assay screening. Comput. Biol. Med..

[18] Hou W., Lu S., Zhao H., Yu Y., Xu H., Yu B., Su L., Lin C., Ruan B.H. (2019). Propylselen inhibits cancer cell growth by targeting glutamate dehydrogenase at the NADP+ binding site. Biochem. Biophys. Res. Commun..

[19] Yin X., Peng J., Gu L., Liu Y., Li X., Wu J., Xu B., Zhuge Y., Zhang F. (2022). Targeting glutamine metabolism in hepatic stellate cells alleviates liver fibrosis. Cell Death Dis..

[20] Zhu M., Fang J., Zhang J., Zhang Z., Xie J., Yu Y., Ruan J.J., Chen Z., Hou W., Yang G. (2017). Biomolecular Interaction Assays Identified Dual Inhibitors of Glutaminase and Glutamate Dehydrogenase That Disrupt Mitochondrial Function and Prevent Growth of Cancer Cells. Anal. Chem..

[21] Liu J., Ghanizadeh H., Li X., Han Z., Qiu Y., Zhang Y., Chen X., Wang A. (2021). A Study of the Interaction, Morphology, and Structure in Trypsin-Epigallocatechin-3-Gallate Complexes. Molecules.

[22] Chen Z., Chen Y., Xue Z., Gao X., Jia Y., Wang Y., Lu Y., Zhang J., Zhang M., Chen H. (2020). Insight into the inactivation mechanism of soybean Bowman-Birk trypsin inhibitor (BBTI) induced by epigallocatechin gallate and epigallocatechin: Fluorescence, thermodynamics and docking studies. Food Chem..

[23] Boodhansingh K., Rosenfeld E., Lord K., Adzick N.S., Bhatti T., Ganguly A., De Leon D.D., Stanley C.A. (2022). Mosaic GLUD1 mutations associated with hyperinsulinism hyperammonemia syndrome. Horm. Res. Paediatr..

[24] Su C., Liang X.J., Li W.J., Wu D., Liu M., Cao B.Y., Chen J.J., Qin M., Meng X., Gong C.X. (2018). Clinical and Molecular Spectrum of Glutamate Dehydrogenase Gene Defects in 26 Chinese Congenital Hyperinsulinemia Patients. J. Diabetes Res..

[25] Senniappan S., Shanti B., James C., Hussain K. (2012). Hyperinsulinaemic hypoglycaemia: Genetic mechanisms, diagnosis and management. J. Inherit. Metab. Dis..

[26] Xu A., Cheng J., Sheng H., Wen Z., Lin Y., Zhou Z., Zeng C., Shao Y., Li C., Liu L. (2019). Clinical Management and Gene Mutation Analysis of Children with Congenital Hyperinsulinism in South China. J. Clin. Res. Pediatr. Endocrinol..

[27] Smith H.Q., Li C., Stanley C.A., Smith T.J. (2019). Glutamate Dehydrogenase, a Complex Enzyme at a Crucial Metabolic Branch Point. Neurochem. Res..

[28] Fang J., Hsu B., Macmullen C.M., Poncz M., Smith T.J., Stanley C.A. (2002). Expression, purification and characterization of human glutamate dehydrogenase (GDH) allosteric regulatory mutations. Biochem. J..

[29] Maurizi M., Rasulova F. (2002). Degradation of L-glutamate dehydrogenase from Escherichia coli: Allosteric regulation of enzyme stability. Arch. Biochem. Biophys..

[30] Lee W., Shin S., Cho S.S., Park J.S. (1999). Purification and characterization of glutamate dehydrogenase as another isoprotein binding to the membrane of rough endoplasmic reticulum. J. Cell. Biochem..

[31] Molven A., Matre G.E., Duran M., Wanders R.J., Rishaug U., Njølstad P.R., Jellum E., Søvik O. (2004). Familial hyperinsulinemic hypoglycemia caused by a defect in the SCHAD enzyme of mitochondrial fatty acid oxidation. Diabetes.

